# Integrated perspectives on colorectal carcinogenesis: molecular pathogenesis, genomic alterations, diagnostic paradigms, therapeutic interventions and AI–driven directions in precision oncology

**DOI:** 10.3389/fonc.2026.1770430

**Published:** 2026-05-14

**Authors:** Sidharth Kumar N., Sai Kiruthiga S., Magesh Ramasamy

**Affiliations:** Department of Biotechnology, Faculty of Biomedical Sciences & Technology, Sri Ramachandra Institute of Higher Education and Research (DU), Chennai, India

**Keywords:** chromosomal instability, colorectal cancer, health care, machine learning, microsatellite instability, quantum computing

## Abstract

Colorectal cancer (CRC) remains a leading cause of cancer-related morbidity and mortality worldwide, with a notable rise in incidence among younger populations and in developing nations. This review provides an integrative perspective on the molecular pathogenesis, epidemiology, classification, diagnosis, and therapeutic strategies in CRC. We emphasize the pivotal role of three major molecular pathways - chromosomal instability (CIN), microsatellite instability (MSI), and CpG island methylator phenotype (CIMP) and their association with tumor heterogeneity and clinical outcomes. The analysis highlights key risk factors including lifestyle-related variables and genetic predisposition, alongside emerging molecular subtypes that influence prognosis and therapeutic decision-making. Advances in non-invasive diagnostics, such as fecal immunochemical testing (FIT) and novel biomarker discovery, demonstrate potential for improving early detection and screening compliance. Preventive strategies encompassing lifestyle modification, chemoprevention, and vitamin D supplementation, coupled with tailored therapeutic interventions including monoclonal antibodies and targeted therapies, offer promising avenues for reducing disease burden. The review also explores recent advances in multi-omics integration and artificial intelligence driven analytics, which have transformed biomarker discovery, subtype classification, and therapeutic prediction in CRC. Emerging applications of machine learning (ML), radiogenomics, and quantum computing further highlight a paradigm shift toward precision oncology. This review underscores the need for precision oncology approaches that integrate molecular profiling, patient stratification, and personalized treatment to enhance health care delivery and clinical outcomes in CRC.

## 1 Introduction

Colorectal cancer (CRC) is the third most commonly diagnosed cancer and the second leading cause of cancer-related mortality worldwide. In 2020, colorectal cancer accounted for around 9.4% of all cancer-related deaths (1). The global incidence of colorectal cancer (CRC) is expected to more than double by 2035 due to the significant increase in identified cases among the elderly, with less developed nations seeing the most severe increase (2).

**Figure 1:**
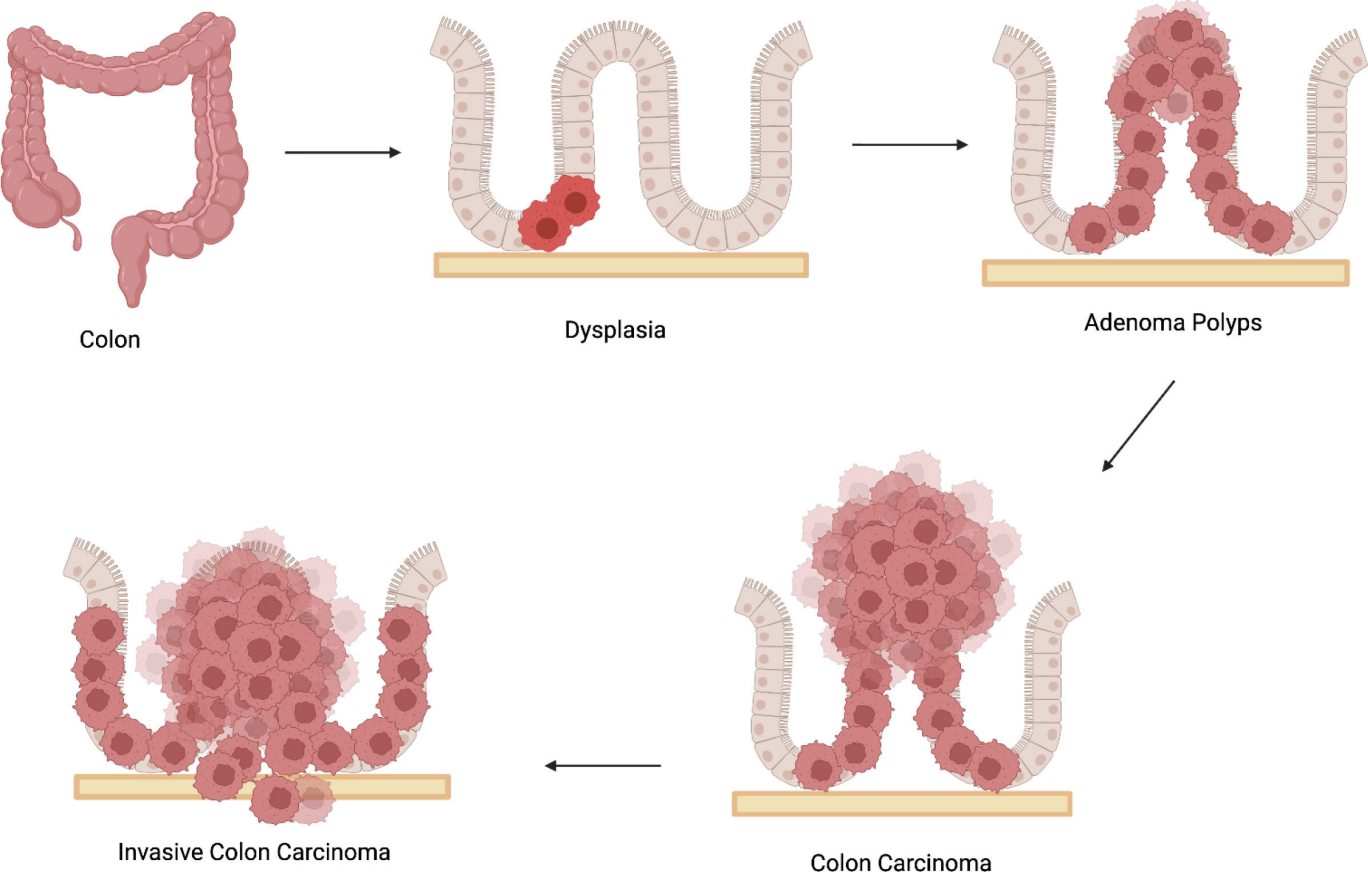
Schematic showing the multistep transformation of normal colon epithelium to adenoma and invasive carcinoma, driven by cumulative genetic mutations, epigenetic dysregulation, and environmental influences.

The aberrant proliferation of glandular epithelial cells in the colon causes colorectal cancer (CRC) that only affects the colon and rectum. Colorectal carcinogenesis follows a progressive transition from normal epithelium to adenoma and subsequently invasive carcinoma, (**Figure 1**). The three primary types of colorectal cancer are sporadic, inherited, and colitis-related. The global incidence of CRC has been steadily increasing in recent years. The chances of developing colorectal cancer is influenced by both hereditary and environmental factors. Furthermore, individuals with Crohn’s disease and ulcerative colitis who have had these conditions for a long period of time are more likely to acquire colorectal cancer (CRC) as they age (3). Numerous studies have shown that family history, chronic inflammation, nutrition and lifestyle are risk factors for colorectal cancer (4).

In addition to improved socioeconomic status, the advancement of civilization and economic growth also results in a shift in food habits known as the “westernization of the lifestyle.” This entails consuming more processed meats, animal fats, refined carbohydrates, and sweets, as well as consuming fewer fruits, vegetables, dietary fiber, and physical exercise. Such a lifestyle is frequently the cause of overweight or obesity (5). Obesity is a well-established risk factor for colorectal cancer (CRC) and contributes to tumor development through mechanisms such as chronic inflammation, insulin resistance, and metabolic dysregulation (6). It has been shown that visceral fat negatively impacts men’s prognosis for colorectal cancer (7). Early diagnosis of colorectal cancer (CRC) is crucial since it typically takes several years to grow. Secondary prevention is also crucial in light of nutrition prevention based on a balanced diet and follow-up exams (8).

## 2 Colorectal cancer development

Despite its genetic diversity, colorectal cancer can arise through a number of distinct methods. For instance, heterogeneity in gene expression across colorectal cancer (CRC) cells results in the accumulation of numerous somatic mutations (9). Therefore, it is thought that among all cancers, colorectal cancer has one of the highest and most remarkable mutational burdens. Colorectal cancer can be broadly classified as either hypermutated (containing more than 12 mutations per 106 bases) or non-hypermutated (having fewer than 8.24 mutations per 106 bases) based on the quantity of somaclonal mutations (10). Parallel attempts to classify colorectal cancer (CRC) according to gene expression patterns have resulted in the development of a unique classification method (10).

**Figure 2:**
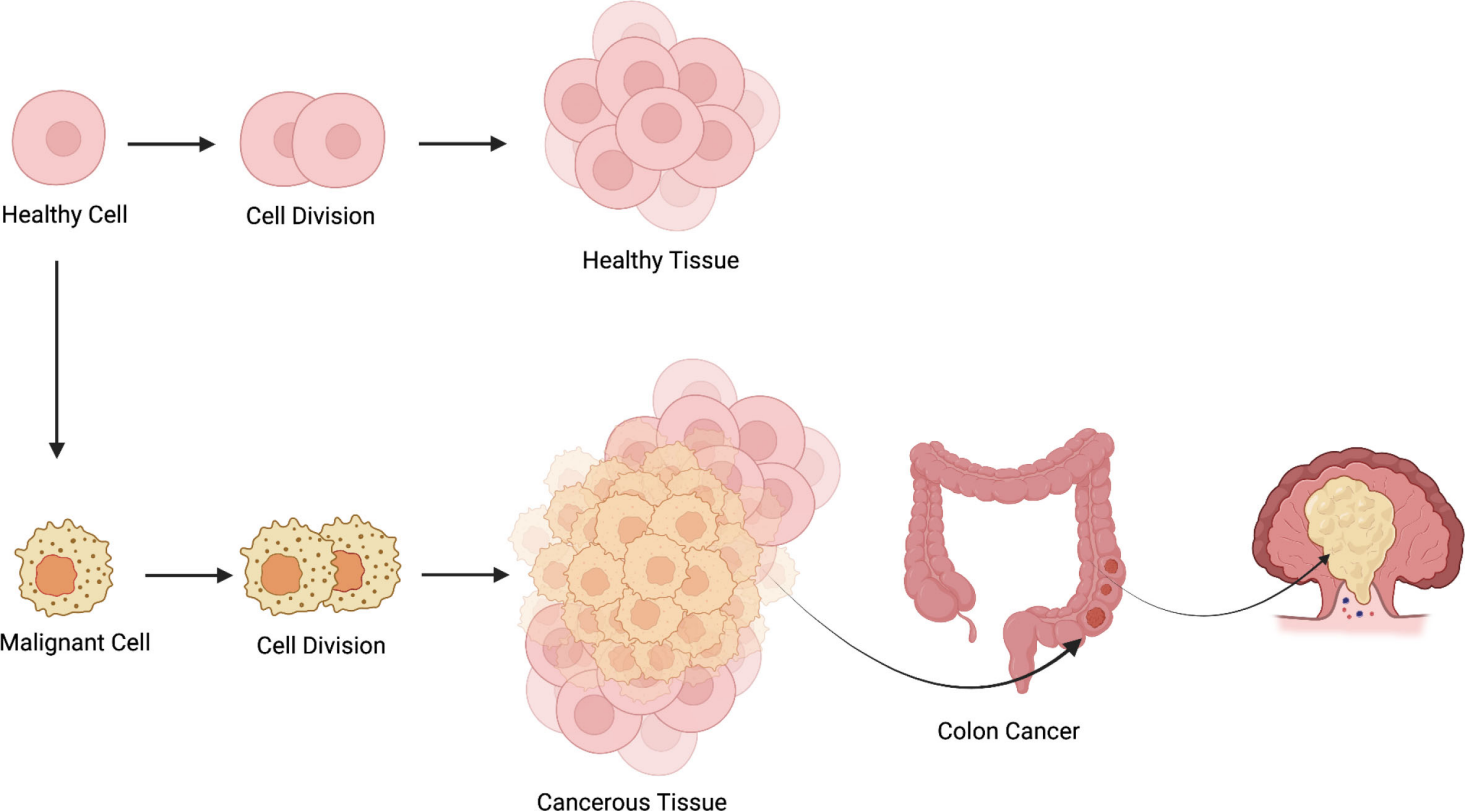
Illustration showing the transformation of healthy colon cells into malignant cells through abnormal cell division, leading to the formation of cancerous tissue and development of colorectal carcinoma.

Colorectal cancer (CRC) develops when epithelial cells experience a variety of genetic and epigenetic changes that enable them to multiply uncontrollably (**Figure 2**) (11). These rapidly growing cells produce a benign adenoma that can develop into cancer and spread by a variety of pathways, including microsatellite instability (MSI), chromosomal instability (CIN), and serrated neoplasia (12–14). The great majority of sporadic CRC cases are caused by the conventional route. Before developing into cancer, a small adenoma develops into a big adenoma. The development of the chromosomal instability (CIN)-positive subtype (CIN-positive) is strongly linked to this route. The National Cancer Institute estimates that 10–15% of sporadic colorectal cancers are caused by this model. It is characterized by the progression of normal cells into sessile serrated adenomas, hyperplastic polyps, and ultimately malignancy (15). Usually, this pathway contributes to the development of the inflammation-associated CpG island methylator phenotype (CIMP-high) subtype. Persistent inflammation induces epithelial alterations that progress from low-grade dysplasia to high-grade dysplasia and eventually invasive carcinoma (15). Benign precursor lesions can be found and treated in all paths, but they are more noticeable in the adenoma–carcinoma and serrated routes (15). Because colorectal cancer develops over a prolonged period, this gradual progression allows for effective secondary prevention through screening and early detection.

## 3 Global epidemiological trends and burden of colorectal cancer

**Figure 3:**
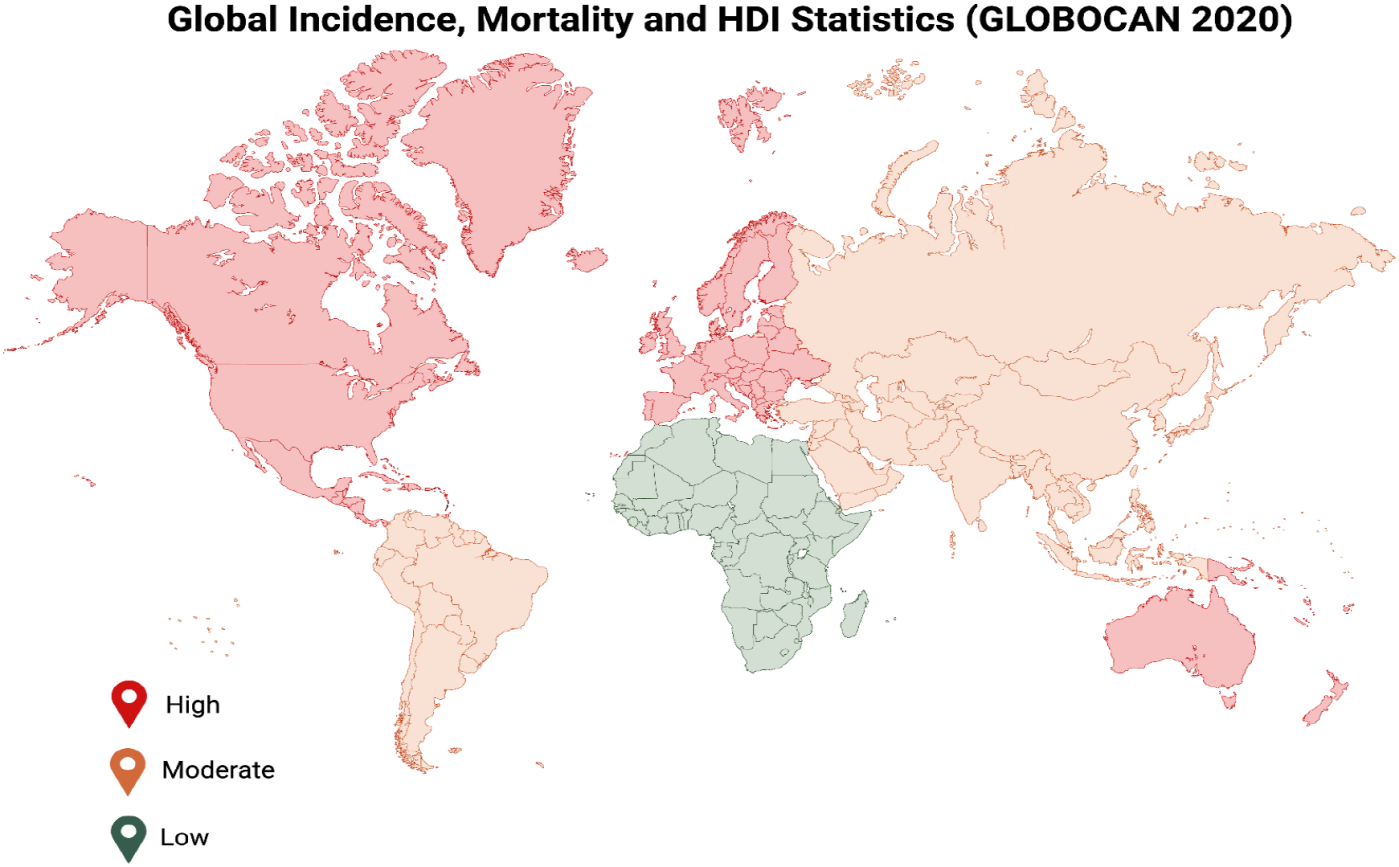
Comparative global statistics illustrating regional disparities in colorectal cancer incidence and mortality rates correlated with Human Development Index (HDI) and lifestyle transitions.

CRC is the second most frequent cancer in women and ranks third in men’s cancer incidence. In 2020, there were more than 1.9 million new cases (16, 17). Colorectal cancer is the second most common cause of cancer-related deaths, accounting for an estimated 900, 000 cancer-related deaths. It is one of the cancers that is becoming more common and makes up 11% of all cancer diagnoses globally (18, 19). According to GLOBOCAN 2020, there is a broad geographic variation in CRC incidence and mortality among various countries (**Figure 3**) (**Table 1**) (19, 20).

**Table 1:** Global incidence and mortality statistics (GLOBOCAN 2020).

Region	Incidence rate (per 100, 000)	Mortality rate (per 100, 000)	HDI category
North America	35.0	12.0	High
Europe	30.5	11.5	High
Asia	17.5	9.8	Medium-High
Africa	8.2	6.1	Low-Medium
South America	15.3	8.5	Medium
Australia	34.0	11.0	High

It has been recognized that the largest increases in CRC incidence and mortality occur in medium and high-human development index (HDI) countries that are adopting the “western” way of life (18). The risk of colon cancer is greater in developed nations. Alcohol, tobacco, red meat intake, obesity, and a sedentary lifestyle are thought to be the main causes of CRC development (19). Therefore, colorectal cancer is a disease of developed countries with a western lifestyle (19, 21). Cancer development is significantly impacted by life expectancy-influencing variables, such as socioeconomic factors (education, wealth, and government spending on health) and health-related habits (exercise, smoking, and obesity). When creating cancer prevention and treatment plans, life expectancy levels must be taken into account (21). A study reported that the incidence of colon cancer rose in 10 out of 36 nations (all in Asia or Europe) between 2007 and 2016, 2006 and 2015, or 2005 and 2014. India saw the biggest rise, followed by Poland (22). The HDI scores of all ten of these nations are medium to high. With the highest HDI ratings, six nations had a decline in the incidence of colon cancer; the United States saw the largest decline, followed by Israel. The incidence of colorectal cancer among individuals aged over 50 years declined in seven countries, including those in North America (1, 23). The incidence of colon cancer among individuals under 50 years of age has increased in eight countries, including the United Kingdom and India. In contrast, Australia, Germany, the United States, Sweden and Canada have reported declining or stable incidence rates among individuals aged 50 years and older, while showing a noticeable increase in cases among those under 50 (18, 23). Recent epidemiological evidence has identified early-onset colorectal cancer (EOCRC), defined as CRC diagnosed in individuals younger than 50 years, as a rapidly emerging global health concern. While the overall incidence of CRC has declined in older populations due to effective screening programs, incidence rates among younger adults have increased significantly over the past two decades across multiple regions, including North America, Europe, and Asia. This trend has been attributed to a combination of lifestyle changes, including increased obesity, sedentary behavior, dietary shifts toward processed foods, and alterations in gut microbiome composition. Importantly, EOCRC often presents at more advanced stages and may exhibit distinct molecular and clinical characteristics compared to late-onset CRC (24). These findings highlight the urgent need for improved risk stratification, early detection strategies, and integration of molecular and computational approaches to identify high-risk individuals and improve clinical outcomes.

The incidence of colon cancer among women increased in 12 of the 36 nations (all in Asia and Europe), while it decreased in seven; the largest increases were seen in India and Slovenia (1). It has been reported that colorectal cancer survival varies according to the stage of diagnosis, leading to a worse survival probability for those diagnosed late (25). Early detection of colorectal cancer is associated with a five-year survival rate of approximately 90%, whereas survival declines to about 13% when diagnosed at advanced stages. Among individuals aged 0–74 years, the cumulative risk of death from colon cancer is estimated at 0.65% for men and 0.45% for women. Worldwide, the age-standardized mortality rate for colorectal cancer is approximately 8.9 per 100, 000 population (17).

It is estimated that by 2030, colorectal cancer (CRC) will account for approximately 2.2 million new cases and 1.1 million deaths worldwide, representing an increase of nearly 60% compared with current estimates. This projected rise is largely attributed to socioeconomic transitions associated with economic development, including lifestyle changes in countries undergoing rapid development and generational shifts in high-income nations. Many studies emphasize that this increase is partly driven by environmental and lifestyle factors, including sedentary behavior, obesity, consumption of highly processed foods, alcohol intake, red meat consumption, and increased life expectancy (26, 27). Comprehensive assessment of global incidence patterns and temporal trends are essential for developing effective prevention and treatment strategies. In addition, public health efforts should emphasize education on CRC risk factors and the implementation of evidence-based screening programs to reduce disease burden. Numerous studies highlight the importance of public health education on colorectal cancer (CRC) risk factors and the implementation of evidence-based screening programs to improve early detection and reduce disease burden (28, 29).

## 4 Classification of colorectal cancer

CRC is often categorized based on the location and histological subtype.

### 4.1 Histological subtypes of CRC

The WHO categorization of colorectal cancer defines the histological subtype (30). There are histological subtypes of colorectal cancer (**Table 2**), such as medullary, adenocarcinoma, signet ringed cell, and mucinous (31). It is possible to differentiate colorectal adenocarcinoma from each of these variants (32). We go into further depth about colorectal adenocarcinoma and three of these variations below.

**Table 2:** Histological subtypes of CRC.

Subtype	Prevalence (%)	Key features	Prognosis
Adenocarcinoma	90%	Glandular epithelial origin	Variable
Mucinous Adenocarcinoma	5–20%	≥50% extracellular mucin	Intermediate
Medullary CRC	4%	Solid growth, inflammatory response	Better
Signet Ring Cell CRC	<2%	Signet ring-like cells	Poor

#### 4.1.1 Adenocarcinoma

Adenocarcinoma is the most common histological subtype of colorectal cancer, accounting for more than 90% of all CRC cases worldwide. These tumors originate from the glandular epithelial cells lining the colorectal mucosa and typically arise through the adenoma–carcinoma sequence involving progressive genetic and epigenetic alterations. Colorectal adenocarcinomas are characterized by gland-forming malignant epithelial cells and exhibit considerable molecular heterogeneity, which influences tumor progression, prognosis, and therapeutic response (33, 34).

#### 4.1.2 Mucinous colorectal adenocarcinoma

Extracellular mucinous pools that comprise at least 50% of the tumor volume are a characteristic of mucinous colorectal adenocarcinoma, the second most prevalent subtype of colorectal cancer. Worldwide, 5–20% of CRC cases are of this subtype (34, 35). “Adenocarcinomas with mucinous features (mucinous adenocarcinoma)” is a term occasionally used to describe cancers that have a significant mucinous component. This kind is characterized by large glandular structures with extracellular mucinous pools (33). Patients with hereditary non-polyposis colorectal cancer (HNPCC) or Lynch syndrome (LS) are more likely to have mucinous adenocarcinomas, that are high level microsatellite instability tumors (35–37).

#### 4.1.3 Medullary CRC

According to estimates, medullary CRC occurs in 4% of all cases (38). The combination of aberrant solid development and an inflammatory response is what defines this subtype (31). MSI-H has a high correlation with medullary cancer (39, 40), often in conjunction with mutations in the BRAF gene, which codes for the serine/threonine-protein kinase that is the proto-oncogene B-Raf.

#### 4.1.4 Signet ring cell CRC

Signet ring cell carcinoma is a rare histological subtype, accounting for less than 2% of all colorectal cancers, and is associated with an aggressive clinical course and poor prognosis (40). Histologically, this carcinoma is characterized by abundant intracellular mucin that displaces the nucleus to the periphery, giving the tumor cells a characteristic signet ring appearance (31) (33). These tumors often present at advanced stages and are associated with diffuse infiltration of the bowel wall, increased metastatic potential, and reduced responsiveness to conventional therapies.

### 4.2 Classification of CRC according to location

Colorectal cancer can also be categorized based on its anatomical location. According to embryological origin, colon cancer is broadly classified into proximal (midgut-derived) and distal (hindgut-derived) tumors. This distinction is clinically relevant because distal colon cancers are often associated with a more favorable prognosis compared with proximal colon cancers (41).

### 4.3 Classification of CRC according to molecular pathway

**Figure 4:**
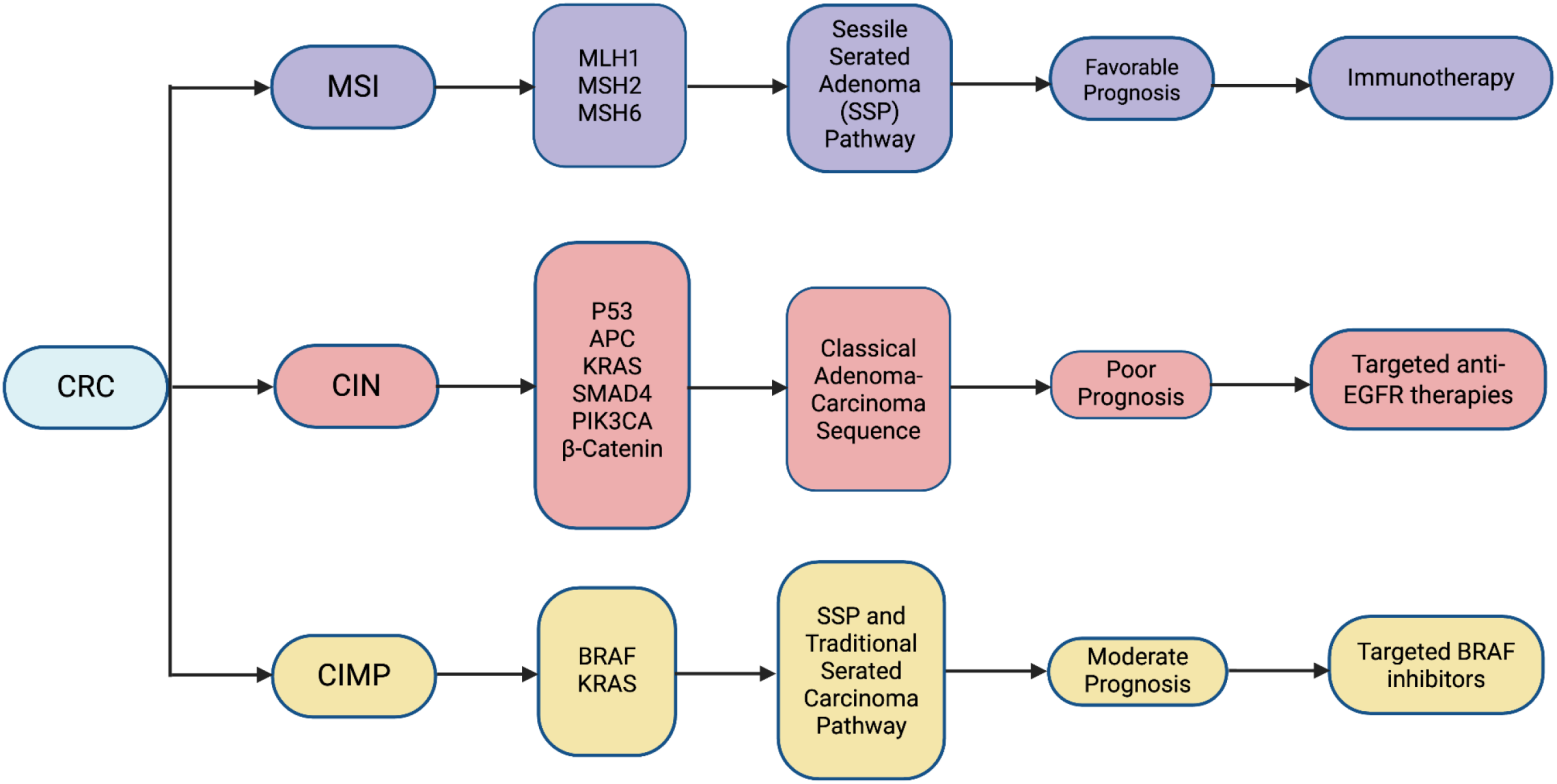
Overview of key molecular mechanisms chromosomal instability (CIN), microsatellite instability (MSI), and CpG island methylator phenotype (CIMP) underlying adenoma-to-carcinoma progression in colorectal cancer.

The importance of classifying molecular pathways has increased, and it is now essential for classifying colorectal cancer. Colorectal cancer (CRC) is caused by mutations that impact genes linked to DNA repair pathways, tumor suppressor genes, and oncogenes. In general, both genetic and epigenetic instability have a major impact on the development and course of colorectal cancer (41, 42). MSI, cytosine preceding guanine (CpG) island methylator phenotype (CIMP), & chromosomal instability (CIN) pathways are three different molecular pathways that are among the pathogenic mechanisms associated with colorectal cancer (**Table 3**)(**Figure 4**) (41).

**Table 3:** Molecular pathways and associated mutations.

Pathway	Key mutations	Frequency (%)	Clinical relevance
CIN	APC, KRAS, TP53, PIK3CA	80–85%	Conventional pathway, high aneuploidy
MSI	MLH1, MSH2, MSH6, PMS2	10–15%	Better prognosis, immunotherapy responsive
CIMP	BRAF, MLH1 hypermethylation	10–15%	Linked with serrated pathway

The CIN pathway, which accounts for approximately 80–85% of colorectal cancers, is characterized by large-scale chromosomal abnormalities including aneuploidy, loss of heterozygosity, and structural rearrangements (43). These alterations result from mutations in key tumor suppressor genes and oncogenes such as APC, TP53, KRAS, and PIK3CA, leading to dysregulated Wnt/β-catenin signaling, uncontrolled cellular proliferation, and genomic instability (44). CIN-positive tumors typically exhibit stepwise progression through the adenoma–carcinoma sequence and are associated with tumor heterogeneity and metastatic potential (45).

In contrast, the MSI pathway arises due to defects in the DNA mismatch repair (MMR) system, primarily involving genes such as MLH1, MSH2, MSH6, and PMS2. Deficient MMR results in accumulation of insertion and deletion mutations in microsatellite regions, generating hypermutated tumors with increased neoantigen burden. MSI-high colorectal cancers often demonstrate improved prognosis and enhanced responsiveness to immune checkpoint inhibitors due to increased immunogenicity. These tumors are frequently associated with Lynch syndrome or epigenetic silencing of MLH1 through promoter hypermethylation (46).

The CIMP pathway represents an epigenetic mechanism characterized by widespread hypermethylation of CpG islands within promoter regions, leading to transcriptional silencing of tumor suppressor genes (47). This pathway is commonly associated with BRAF mutations and serrated precursor lesions and contributes to altered gene expression, tumor progression, and molecular heterogeneity (48). Collectively, these molecular pathways highlight the complex interplay between genetic and epigenetic alterations in colorectal cancer and provide critical insights for biomarker development, targeted therapies, and precision oncology.

## 5 Multifactorial etiology of colorectal cancer: genetic, molecular, and lifestyle influences

The development of colorectal cancer (CRC) is commonly described through the sequential phases of initiation, promotion, and progression. The process begins with irreversible genetic damage that renders intestinal mucosal cells susceptible to neoplastic transformation (49). During the promotion phase, genetically altered cells undergo clonal expansion, resulting in abnormal cellular proliferation. During the progression stage, these lesions acquire additional genetic alterations that confer aggressive characteristics and metastatic potential, ultimately leading to malignant transformation. The existence of a benign precursor lesion, known as a polyp (defined as an abnormal growth on the colon mucosa developing in its lumen), is an essential component of the majority of CRC carcinogenesis processes. Adenomatous polyps and serrated polyps, which are the direct progenitors of the majority of malignancies, are another form of lesion seen in the lumen of the large intestine (50). Compared to non-advanced adenomas (1%), advanced adenomas (≥1 cm in diameter), with or without variety, have a much greater chance of cancer development (between 30% and 50%). Furthermore, the greater transition rates to cancer, which rise with age, are characterized by advanced adenomas (51, 52). The other gut wall alterations, like polished polyps, are a collection of diverse lesions that include mixed polyps, hyperplastic polyps, classic serrated adenomas, and sessile serrated adenomas (53). They combine the toothed morphological appearance of hyperplastic polyps and dysplastic features of adenomas, and these changes are precursors to approximately 10–15% of sporadic CRC. However, the most common lesion present in the gut is a hyperplastic polyp (80–90%) (54). Hyperplastic polyps, particularly large lesions and those located in the proximal colon, can progress to colorectal cancer through the serrated pathway via traditional serrated adenomas or sessile serrated adenomas (55). The carcinogenesis of colorectal cancer is unquestionably a gradual process that begins with mild inflammation, progresses to adenomatous polyps in the epithelium, and ends with adenocarcinoma. Furthermore, the process takes 10 to 15 years, but may go more quickly in specific circumstances, such as Lynch syndrome patients, and is fueled by the accumulation of mutations and genetic alterations (56) (57).

Patients suffering with inflammatory bowel disease, especially ulcerative colitis, may also develop colorectal cancer (CRC) along the inflammatory route. These people have colon cancer, low-grade dysplasia, high-grade alterations that lead to neoplastic transformation, and dysplasia that persists indefinitely.

The CIN pathway is responsible for between 70 and 85 percent of all CRC incidences (44, 58, 59). In addition to chromosomal instability (CIN) and microsatellite instability (MSI), colorectal cancer can also develop through a third pathway associated with the CpG island methylator phenotype (CIMP) (60, 61). It is linked to the loss of TP53 and p16 functions, the V300E mutation in the BRAF gene, and hypermethylation of many gene promoters, including MLH1. These conditions result in the silence of suppressor genes, which disrupts the functioning of the MMR system and leads to MSI and hypermutation. This mechanism is most frequently seen in women in the proximal portion of the colon when serrated architectural lesions occur (62).

CRC is a non-homogeneous disease entity, as was previously stated. The location, level of histological malignancy, and kind of tumor vary from case to case. The multilayer molecular intricacy, however, is the most fascinating aspect. Four molecular subtypes of colorectal cancer (CMS) were established by the 2015 CRC Subtyping Consortium consensus: CMS1 (MSI-immune activation), CMS2 (canonical), CMS3 (metabolic), and CMS4 (mesenchymal). Because each subtype has a unique clinical history and reacts differently to biological and chemotherapeutic treatments, the categorization has practical significance. In addition to being a useful predictive and prognostic tool, this may assist choose the best, customized treatment plan for each patient. The use of these events in molecular screening for colorectal cancer is arguably the most promising (63).

On the so-called serrated lesion pathway, which accounts for 10% of all colorectal cancers, adenomatous polyps are replaced by serrated polyps to become serrated adenocarcinomas. Although several genes are epigenetically silenced and the BRAF mutation is present in these cancers, the APC gene is not implicated as it is in other pathways. Microsatellite instability (MSI), which results from the interruption of DNA repair genes, is another factor that contributes to colorectal cancer (63). Adenomas and cancer can result from a combination of environmental exposure and an intrinsic genetic vulnerability. However, the majority of colorectal cancers are sporadic, meaning that people do not have a hereditary load, and lifestyle and environmental variables are associated with the development of this disease. Furthermore, prolonged exposure to pollutants may encourage oxidative stress. By gradually accumulating somatic mutations, oxidative stress can increase DNA damage and cause instability in the genome.

## 6 Characteristic symptoms and disease presentation in colorectal cancer

Colorectal cancer (CRC) may be taken into consideration when specific lower gastrointestinal (GI) symptoms manifest. Medical practitioners can utilize guidelines published by the National Institute for Health and Professional Excellence to assess if a patient is at a high risk of colorectal cancer. Changes in bowel habits, unexplained weight loss, iron-deficiency anemia, rectal bleeding, abdominal mass, and abdominal pain are all signs of probable colorectal cancer (CRC) and should be submitted to further testing (64). However, some symptoms, such deep vein thrombosis and unexplained appetite loss, should be noted as they are not site-specific. A referral to the suspected cancer pathway or an urgent investigation for these symptoms may be made after a review of other symptoms, indications, or outcomes that may help identify which cancer is most likely to be explored (65).

The value of symptoms in identifying colorectal cancer has been assessed in a number of research. They exhibit isolated symptoms or indicators that are not very useful for determining the specificity and sensitivity of colorectal cancer. Additionally, positive as well as negative likelihood ratios (PLR and NLR) reveal that the presence or absence of symptoms has little influence on the odds of diagnosing colorectal cancer (65–67). Nonetheless, according to a number of standards, colonoscopies are performed on patients who exhibit bowel signs and symptoms that might indicate colorectal cancer in clinical practice (68). According to certain studies, a palpable abdominal mass during examination, a report of deep red rectal bleeding, rectal bleeding along with weight loss, or a change in bowel motions, for instance, may improve the diagnostic sensitivity and specificity to colorectal cancer (66).

In terms of CRC therapy, those who were diagnosed prior to experiencing CRC symptoms (or these were the initial symptoms) and whose condition was identified early on have a significantly better prognosis. Therefore, any concerning symptoms that might indicate colorectal cancer should prompt the patient to contact a physician immediately and undergo colon diagnostic testing (69).

## 7 Interplay of hereditary, dietary, and environmental factors in colorectal cancer susceptibility

**Figure 5:**
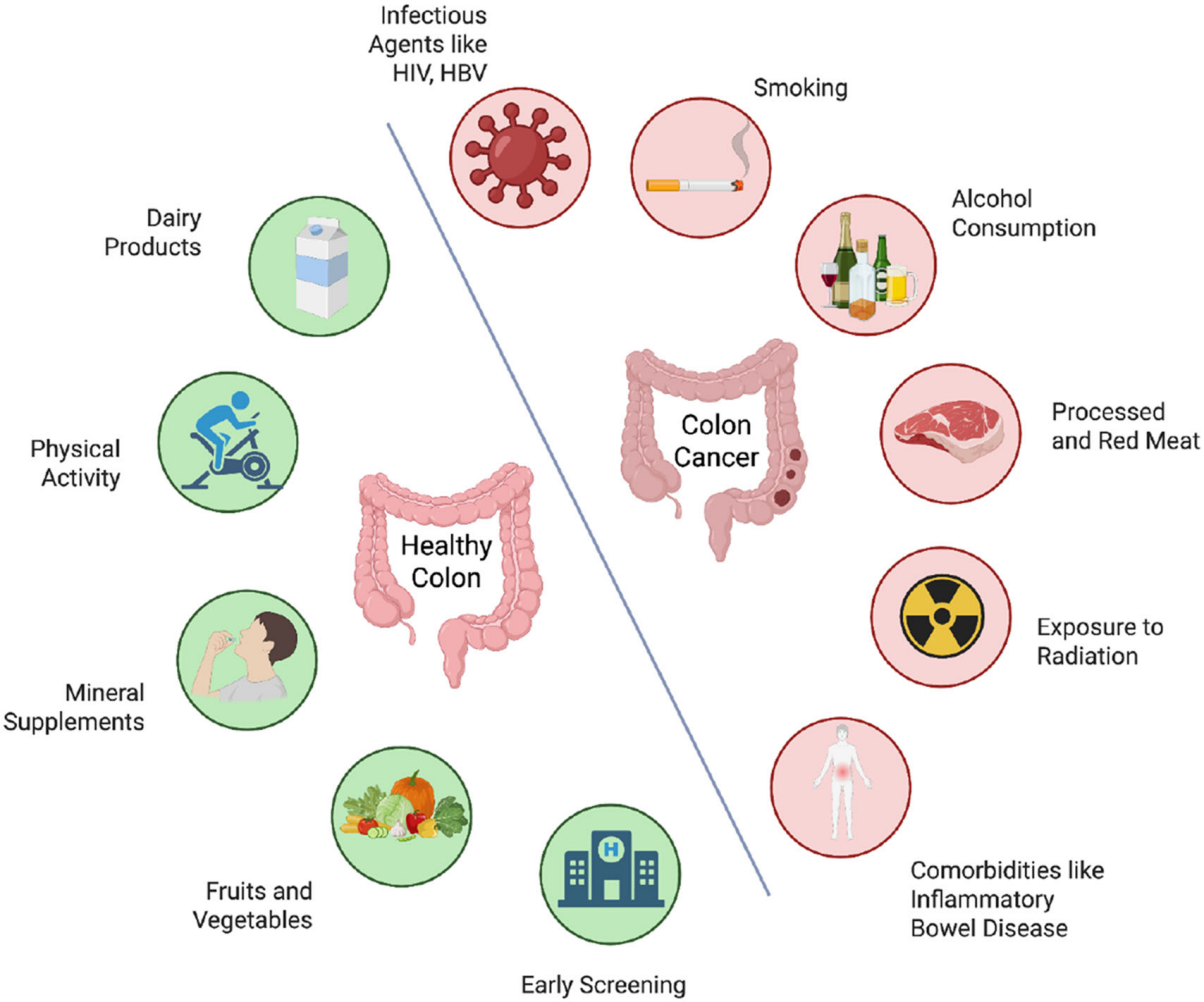
Illustration contrasting environmental and genetic risk factors promoting colorectal cancer with lifestyle and dietary components that help maintain a healthy colon.

The occurrence of CRC, which is regarded as a heterogeneous disease, is connected with a number of risk factors (42, 70). The etiology and risk factors for colorectal cancer (CRC) are largely influenced by both hereditary and environmental factors (**Figure 5**) (71), whereas a slightly elevated risk of colorectal cancer is linked to other risk factors.

### 7.1 Inherited genetic risk

Hereditary factors are one of the CRC risk factors that cannot be controlled (70). Several inherited genetic syndromes are associated with an increased risk of CRC. Familial adenomatous polyposis (FAP) and Lynch syndrome (LS) are among the most common hereditary conditions linked to CRC (24, 28, 56). Together, these hereditary syndromes account for approximately 5% of all colorectal cancer cases.

### 7.2 Personal and family history

Adenomatous polyps and the personal and family history of CRC patients are linked to a higher likelihood of developing synchronous and metachronous primary CRC. Furthermore, an elevated risk of colorectal cancer is linked to a personal history of IBD (Crohn’s disease and ulcerative colitis) (70).

### 7.3 Race and ethnicity

CRC survival varies greatly and may be influenced by race and ethnicity. For instance, compared to Hispanic and white Americans, Native Americans and African Americans in the USA are more likely to acquire colorectal cancer (CRC) and have poorer survival rates at all stages of the disease (72).

### 7.4 Sex

Men have a somewhat greater risk of colorectal cancer (CRC) than women do. In every country, males are 1.5 times more likely than women to acquire colorectal cancer (CRC) at any age. Additionally, women are more likely than males to develop right-sided colon cancer, which exhibits a more aggressive phenotype than left-sided colon cancer (72, 73).

### 7.5 Age

Another uncontrollable risk factor for CRC is age. More than 90% of CRC cases involve people who are 50 years of age or older. People over 60–79 years have a >50-fold greater incidence risk of colorectal cancer (CRC) than people under 40.

### 7.6 Comorbidities

Comorbidities that increase the risk of colorectal cancer (CRC) include diabetes mellitus/insulin resistance, uncontrolled acromegaly, cholecystectomy, long-term immunosuppression following kidney transplantation, cystic fibrosis, childhood cancer survivors who received abdominal irradiation, and patients with cancer of the prostate undergoing androgen deprivation therapy (72).

### 7.7 Environmental factors and lifestyle

Strong correlations between lifestyle and environmental variables and the development of colorectal cancer have been documented. Poor medical treatment combined with a low socioeconomic position raises the risk of colorectal cancer. CRC risk is also linked to nutritional behaviors such as eating a lot of meat, especially red and processed meat, and eating a diet rich in fat and low in fruits, vegetables, and fiber. Furthermore, it has been noted that obesity, excess body weight, and a lack of physical exercise raise the risk of colorectal cancer. Heavy alcohol use and cigarette smoking may also be linked to an increased risk of colorectal cancer (70).

## 8 Prevention and risk-reduction approaches for colorectal cancer

Dietary variables may be linked to the increasing prevalence of colorectal cancer (CRC), according to important and new discoveries on the molecular pathways underlying the disease’s development. Unfortunately, because of its extremely complicated physics, the evidence to date is insufficient. Furthermore, a strong association between colorectal cancer and gut microbiota has been hypothesized. Once more, the development and spread of colorectal cancer are linked to changes in the balance of the intestinal microbiota and dietary practices. However, altering the gut microbiota is a proven way to boost therapeutic efficacy and reduce side effects from CRC treatment. To treat colorectal cancer (CRC) effectively and pinpoint the main obstacles for a long-term solution, a number of difficult problems, such as cause, diagnosis, therapy, and management, must be addressed (73).

### 8.1 Primary prevention

In a population that is otherwise healthy, colorectal cancer is prevented by primary preventive strategies. It is expected that the recent drop in CRC incidence and death in the US will continue if exposure to risk factors keeps going down at its current pace. Incidence rates may decrease far more if risk factor removal picks up speed. While it is possible to alter risk factors for many cancers, doing so often results in a long-term decrease in incidence rates rather than a short-term one. This makes it difficult to monitor and evaluate the impact of changes in the prevalence of CRC risk factors on incidence rates.

Numerous potentially changeable characteristics have been found via epidemiological research, which offers both opportunities and difficulties for primary prevention. Reducing cigarette smoking, excessive alcohol use, being overweight or obese, and consuming a lot of red and processed meat can all help avoid colorectal cancer. Regular aspirin use, physical exercise, and hormone replacement treatment have also been shown to lower the risk of colorectal cancer (CRC). Furthermore, some research suggests that eating whole grains and milk may help prevent colorectal cancer (74–77).

### 8.2 Secondary prevention

In contrast to other malignancies, colorectal cancer often develops slowly over a period of years to decades when the normal colon epithelium changes into an adenoma. Due to the slow evolution of the adenoma–carcinoma sequence, colonoscopy is often a viable method for secondary prevention of colorectal cancer (CRC) since it can identify and remove adenomas, hence diagnosing CRC at an earlier stage. Simultaneously, being treated allows for more successful secondary death prevention. Stool tests like FOBTs and endoscopic inspections of the large intestine, especially flexible sigmoidoscopy and colonoscopy, are the most often used screening techniques. Though computed tomography (CT), blood or urine tests, capsule endoscopy, and other stool-based (including DNA-based) diagnostics have been proposed, their usage has been restricted due to concerns over their high cost and diagnostic effectiveness. Moreover, CT is known to have negative effects. However, scientists worldwide are searching for novel biomarkers, such blood-based “omics signatures, ” which might greatly expand the range of noninvasive or minimally invasive CRC screening tests already available (3).

### 8.3 Tertiary prevention

Numerous chances for tertiary prevention are suggested by the growing epidemiological data that some of the major risk factors for developing colorectal cancer also affect survival outcomes. For instance, it is believed that a cancer diagnosis presents a wonderful “teachable moment” that may be used to encourage patients to make the required lifestyle changes, even if it is acknowledged that it is difficult to persuade people to change dangerous lifestyle variables.

Two important modifiable risk factors for CRC are cigarette smoking and heavy alcohol consumption. Both appear to be linked to lower survival rates (78), highlighting the urgent need to encourage and assist patients to achieve necessary lifestyle changes. Although the exact mechanism connecting these specific risk factors to lower survival rates has not been established, it is thought that higher rates of surgical complications, worse responses to chemotherapy and radiation, and nicotine-induced suppression of cancer cell apoptosis and enhancement of cell migration all contribute to lower survival rates (79).

However, studies indicate a connection between physical activity and positive cancer outcomes, including decreased fatigue, improved quality of life, and longer survival (80). Numerous theories have been put out to explain this association, including oxidative stress, metabolic dysregulation, chronic inflammation, lower visceral and total body fatness, and improved immunological function linked to physical exercise. Comprehensive randomized controlled trials (RCTs) evaluating the immediate and long-term impacts of various forms of physical exercise during and after the postoperative phase are necessary for the advancement of this field of research. Short- and long-term physical activity programs tailored to each patient’s requirements and condition should be implemented in conjunction with cancer therapy and monitoring, as demonstrated by prior RCTs that demonstrated a favorable association between outcomes and specific physical activity-based treatments. Patients with colorectal cancer (CRC) should engage in 17.5 to 35 metabolic equivalent task (MET) hours of physical activity per week to reduce mortality by 30 to 40%. A minimum of 3.5 MET-hours of physical exercise per week is recommended for people with reduced physical capacity. Increasing exercise levels after being diagnosed with colorectal cancer (CRC) decreased overall mortality (p for trend = 0.003) and cancer-specific mortality], according to a research including 573 women with stage I and III CRC. However, there was no statistically significant correlation between physical activity and overall survival among patients with metastatic colorectal cancer (CRC) in a prospective cohort trial involving over 1200 patients (81–83).

There is now a lot of research being done on the possible application of chemoprevention in tertiary prevention. Low-dose aspirin usage, which is already associated with a decreased risk of colorectal cancer, has been reported in several observational studies to improve survival rates after receiving a colorectal cancer diagnosis. It could do this via inhibiting COX. To find and learn more about a potential role for aspirin in tertiary prevention, studies of other non-COX routes have been started. To assess additional speculative evidence that metformin can improve survival rates among patients with colorectal cancer, RCTs are also necessary. It is believed that significant errors, including immortal time bias, in certain significant pharmacoepidemiological studies led to the widespread prescription of beta-blockers and other medications to treat comorbidities in CRC patients, which have now been shown to be ineffective in this population (84–86).

Additionally, vitamin D insufficiency, which is frequently observed in patients with colorectal cancer, has been linked to a significantly decreased likelihood of survival, according to epidemiological study. Numerous explanations have been put out to explain this association, including the effects of vitamin D on immunomodulation, proapoptosis, and antiangiogenetics. Vitamin D supplementation has a clinically meaningful impact on CRC survival outcomes, according to a comprehensive review and meta-analysis of randomized controlled trials. High-dose vitamin D supplements increased progression-free survival for patients with metastatic colorectal cancer, according to a recent randomized phase II research. To evaluate the function of vitamin D supplementation in tertiary prevention, more RCTs are needed (87, 88).

## 9 Multi-modal approaches to the early detection and diagnosis of colorectal cancer

**Figure 6:**
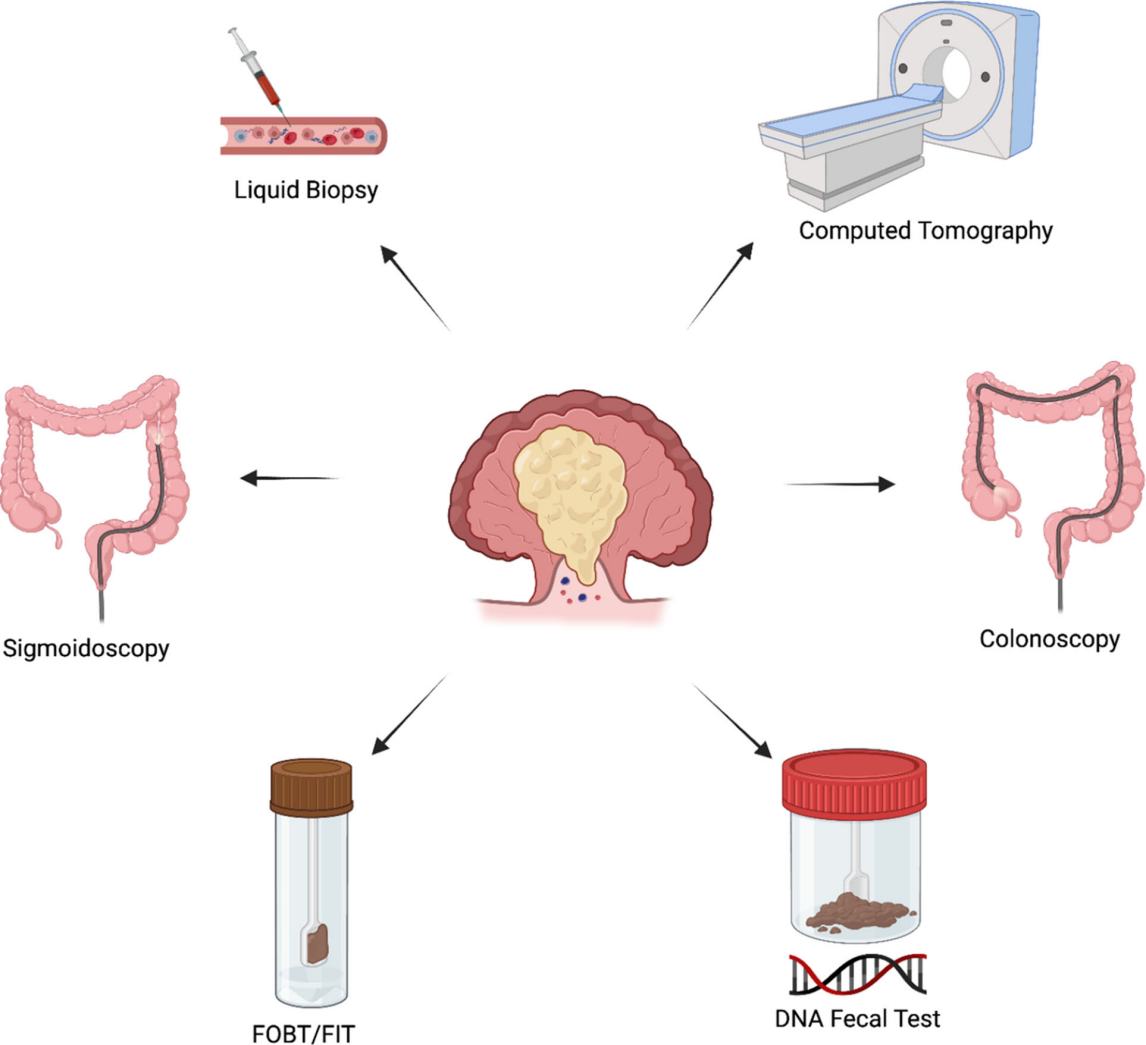
Flowchart summarizing standard diagnostic and screening modalities for colorectal cancer for early detection and effective prevention.

Primary care doctors review the patient’s medical history and do a physical examination of the abdomen in order to identify colorectal cancer (CRC). When colorectal cancer is suspected, the patient is directed to a gastrointestinal clinic based on the results of the physical examination and subject evaluation. Before selecting the best optical and/or imaging diagnostic technique, the doctor should verify the patient’s family history and evaluate risk factors. Numerous screening initiatives, whether structured, opportunistic, or pilot, are in existence worldwide and provide an additional means of identifying colorectal cancer (89). The participation percentage ranged from 16.1% to 68.2%, despite the fact that there were more sessions. Most program members are between the ages of 50 and 75, and screening methods differ significantly depending on colonoscopy capabilities, financial resources, and protocols developed during the research stage. In Western countries, where colorectal cancer is more common, screening programs are more frequently implemented with a specific type of test. The majority of screening diagnostic methods include digital rectal examination (DRE), flexible sigmoidoscopy (FS), optical colonoscopy (OC), guaiac fecal occult blood testing (gFOBT), and fecal immunochemical testing for hemoglobin (**Table 4**) (**Figure 6**).

**Table 4:** Conventional screening and diagnostic modalities for colorectal cancer.

Test	Sensitivity (%)	Specificity (%)	Recommended population
FIT	79	94	Average risk, >50 years
gFOBT	50–60	85	Average risk, >50 years
Colonoscopy	95	95	High-risk and general screening
Flexible sigmoidoscopy	80	90	Average risk

A thorough assessment of the family history of cancer in first, second, and third-degree relatives is essential to obtain complete information for the diagnosis process. The following should be included in the data collected: kind of cancer, medical history, ethnicity, age at when cancer was diagnosed, current age or age and reason of death, and relative consanguinity. Studies show that those with FDRs who already have CRC are at a higher risk of developing CRC (90). Additionally, the screening procedure for the detection of colorectal cancer is influenced by the quantity and quality of relatives. The patient is checked by colonoscopy every 5–10 years or by FIT every 1–2 years starting at age 40–50, or 10 years before the FDR age of diagnosis, if there was one FDR with colorectal cancer or several FDRs with advanced adenoma. If a patient has several FDRs or SDRs with colorectal cancer (CRC) or polyps, or if more than two FDRs with CRC are in their family history, a colonoscopy should be performed every five years starting at age 40 or ten years before the FDR diagnosis (91). Other details should include such situations like non-paternity or delivery from sperm/egg donors. MUTYH-associated polyposis, Lynch syndrome, familial adenomatous polyposis, hamartomatous polyposis, and other syndrome-specific characteristics are assumed to be part of a family history of probable colorectal cancer (CRC). When the patient is between the ages of 20 and 25, surveillance should begin, and they should adhere to high-risk criteria. A palpable abdominal tumor, peripheral lymphadenopathy, hepatomegaly, and ascites should also be watched for in patients with probable colorectal cancer.

The preferred screening test in primary care is the fecal occult blood test. Although not always advised for all symptomatic individuals, it has been suggested that those with low-risk bowel problems be sent. Immunochemical-based (FIT) or high-sensitivity guaiac-based (HSgFOBT) tests are advised for CRC screening and occult bleeding detection (**Table 5**) (91).There are a number of restrictions that must be adhered to prior to testing since gFOBT is not unique to human hemoglobin and various meals or medications may alter the test’s results. For patients with low-risk CRC symptoms, FIT is advised in lieu of gFOBT as it quantifies the quantity of human-specific hemoglobin in a fecal sample. According to NICE recommendations, it should be used in general care or for screening for suspected colon cancer in patients with iron deficiency anemia and inexplicable changes in bowel movements, including those aged 60 and over, even if they do not have iron deficiency. In order to make decisions about regular or urgent therapy, FIT may help safely and objectively predict each patient’s risk of developing colorectal cancer (CRC) when used in conjunction with clinical examination. In terms of detection rate, positive predictive value, and participation rate, FIT also performs better than gFOBT. With a cut-off of around 10 μg Hb/g feces, quantitative FIT can reliably exclude colorectal cancer (CRC) and reduce the need for colonoscopies in 75–80% of patients with symptoms, according to recent meta-analyses. The proposal that individuals with unexplained symptoms but no rectal bleeding who do not meet the criteria for suspected colorectal cancer should routinely undergo FIT in primary care is currently unsupported by enough data. However, new research indicates that FIT is quite successful in classifying individuals with low-risk CRC symptoms (92, 93).

**Table 5:** Stage-specific diagnostic performance of artificial intelligence–assisted detection methods in colorectal cancer.

Method	Target Lesion	Sensitivity (%)	Specificity (%)	Clinical significance
AI-assisted colonoscopy	Adenomas (precancerous polyps)	90–95	85–92	Improves early precancer detection
AI-assisted colonoscopy	Early-stage CRC (Stage I–II)	88–94	90–96	Enables early diagnosis and intervention
AI-assisted colonoscopy	Advanced CRC (Stage III–IV)	92–98	92–97	Improves advanced tumor detection
Multi-target stool DNA (Cologuard)	Early-stage CRC	92	87	Non-invasive early detection
FIT	Early-stage CRC	74–88	90–96	Population screening tool
Liquid biopsy (ctDNA)	Early-stage CRC	70–90	>95	Emerging biomarker

The diagnostic performance of AI-based colorectal cancer detection varies depending on lesion stage and type. AI-assisted systems demonstrate particularly high sensitivity for detecting adenomas and early-stage colorectal cancer, which is critical for early intervention and improved survival outcomes. Stage-specific evaluation provides a more accurate representation of diagnostic performance and clinical utility compared to aggregated metrics.

Recent advances in non-invasive colorectal cancer screening have significantly improved early detection and patient compliance. Multi-target stool DNA testing, such as Cologuard, combines detection of fecal hemoglobin with molecular analysis of DNA mutations and methylation markers associated with colorectal carcinogenesis, including alterations in KRAS and aberrant methylation of BMP3 and NDRG4 genes. These tests demonstrate higher sensitivity for detecting colorectal cancer and advanced adenomas compared to conventional fecal immunochemical tests (FIT), particularly in early-stage disease (94). Advanced FIT-based screening approaches have also improved diagnostic accuracy through quantitative hemoglobin measurement and optimized cutoff thresholds, enhancing early lesion detection. In addition, emerging non-invasive approaches such as circulating tumor DNA (ctDNA)-based liquid biopsy show promise for early detection, monitoring treatment response, and identifying minimal residual disease. These non-invasive screening strategies offer effective alternatives to colonoscopy, improving screening accessibility, patient compliance, and early diagnosis (95).

## 10 Contemporary therapeutic modalities in colorectal carcinogenesis

An important component of increasing CRC patients chances of survival is early diagnosis (96). If identified early, before metastases, some cancer types may be curable (74, 97). Chemotherapy, surgery, and radiation therapy are often the conventional methods of treating CRC, or a combination of these for late stages (**Table 6**) (75). The location, size, degree of cancer metastasis, and patient health state are among the tumor-related factors that influence the multimodal strategy used to treat colorectal cancer (76). Patients now have a variety of treatment approaches that have generally emerged, including radiotherapy, neoadjuvant chemotherapy, palliative chemotherapy, laparoscopic surgery for early-stage primary colorectal cancer (CRC), and more aggressive resection of mtCRC (such as pulmonary and liver metastases). Surgical resection is the main treatment for patients with locally localized, early-stage colorectal cancer that may be cured (77); However, neoadjuvant chemotherapy and/or radiation treatment may be administered before to or after surgery, contingent on the disease’s stage. Of all the solid tumor types, only those with colorectal cancer (CRC) can benefit from surgically excising distant metastases from organs such as the liver and lungs.

**Table 6:** Summary of treatment available for CRC.

Stage	Recommended treatment	Survival benefit
Stage I–II	Surgical resection	High (70–90%)
Stage III	Surgery + adjuvant chemotherapy	Moderate (50–70%)
Stage IV	Systemic chemotherapy ± targeted therapy, palliative care	Low (<15%)

People with incurable mtCRC may have a better chance of survival if they get systemic chemotherapy. In colorectal cancer (CRC), monoclonal antibodies against EGFR and VEGF are commonly used in combination with chemotherapy to stop tumor growth and angiogenesis. To improve quality of life and prolong longevity, a palliative systemic approach is used for non-surgical CRC cases (patients with late-stage mtCRC). Probiotics, agarose macrobeads, gold-based drugs, and anti-inflammatory drugs are among the alternative therapies now being studied as means of enhancing treatment effectiveness and reducing the side effects of traditional chemotherapy. Nevertheless, despite the progress in treating colorectal cancer, late identification of the disease and the safety and efficacy of chemotherapy remain major obstacles to treatment success (98).

## 11 Artificial intelligence–driven multi-omics integration and computational oncology in colorectal cancer: established applications, emerging technologies, and future perspectives

### 11.1 Established clinical applications of artificial intelligence in CRC

Recent advances in artificial intelligence have enabled clinically applicable solutions for colorectal cancer diagnosis, molecular characterization, and prognostic prediction. AI-assisted colonoscopy systems using convolutional neural networks (CNNs) have demonstrated high sensitivity and specificity for real-time polyp detection, significantly improving adenoma detection rates and reducing missed lesions. In addition, AI-driven digital pathology platforms can automatically analyze histopathological slides to classify tumor grade, detect lymph node metastasis, and assess tumor microenvironment composition, improving diagnostic accuracy and clinical decision-making (99). Radiogenomic approaches integrating imaging data from CT, MRI, and digital pathology with genomic and transcriptomic information have enabled non-invasive prediction of molecular alterations such as KRAS, BRAF, and microsatellite instability (MSI) status. These clinically validated applications demonstrate the integration of artificial intelligence into routine colorectal cancer diagnostics and precision oncology workflows.

### 11.2 Emerging artificial intelligence applications in multi-omics integration and therapeutic discovery

Emerging AI-driven approaches are transforming colorectal cancer research through integration of multi-omics datasets, including genomic, epigenomic, transcriptomic, proteomic, metabolomic, and microbiome data, enabling improved biomarker discovery and enhanced mechanistic understanding (100). Machine learning models such as Random Forest, Support Vector Machine, Gradient Boosting, and Deep Neural Networks have enabled identification of novel diagnostic and prognostic biomarkers, molecular subtype classification, and therapeutic response prediction (101). Advanced deep learning architectures, including autoencoders, graph neural networks, and transformer-based fusion models, facilitate integration of heterogeneous omics data and enable identification of complex molecular interactions and regulatory networks underlying tumor progression (102).

**Figure 7:**
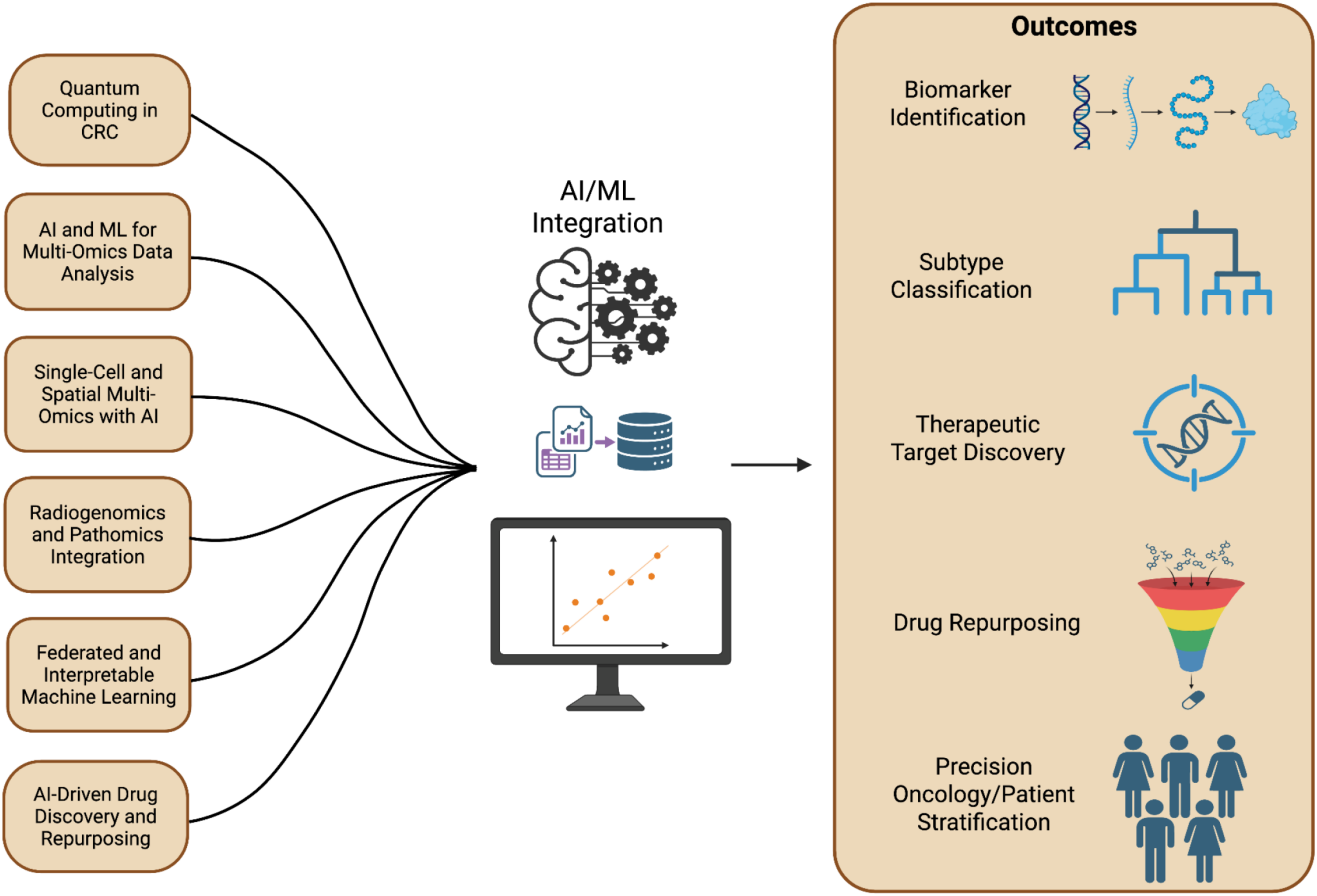
AI/ML integration in colorectal cancer (CRC). Advanced computational approaches including multi-omics, radiogenomics, and AI-driven modeling enable biomarker discovery, subtype classification, therapeutic target identification, and precision oncology applications.

Single-cell and spatial multi-omics technologies integrated with AI algorithms have further enhanced understanding of tumor heterogeneity and tumor microenvironment dynamics (103). AI-driven clustering and trajectory inference methods enable identification of cancer stem cell populations, immune cell infiltration patterns, and metastatic signatures at high spatial resolution (104). Additionally, AI-based drug discovery platforms integrate transcriptomic signatures with chemical–gene interaction databases to identify repurposable drugs and novel therapeutic targets (**Figure 7**) (105). Generative AI and reinforcement learning models are also being used to design new compounds targeting dysregulated colorectal cancer pathways such as Wnt/β-catenin, PI3K/AKT, and TGF-β signaling pathways.

Federated learning has emerged as an important approach enabling collaborative multi-institutional model development without compromising patient privacy. Interpretable AI methods, including SHAP and explainable neural networks, further enhance transparency and clinical reliability of predictive models, facilitating translation of AI-driven approaches into clinical practice (106, 107).

### 11.3 Future and theoretical applications: quantum computing and next-generation artificial intelligence

Quantum computing represents a promising future direction with the potential to revolutionize colorectal cancer research and precision oncology. Quantum machine learning and hybrid quantum-classical models offer enhanced computational capabilities for analyzing high-dimensional multi-omics datasets, enabling faster feature selection, biomarker discovery, and predictive modeling (108). Quantum algorithms such as variational quantum circuits and quantum kernel methods have demonstrated early potential in histopathological image classification and molecular feature optimization.

Although quantum computing applications in colorectal cancer remain largely experimental, continued advancements in quantum hardware and algorithms are expected to significantly accelerate multi-omics analysis, drug discovery, and predictive modeling. Integration of quantum computing with artificial intelligence may enable unprecedented computational efficiency and enhance precision oncology by improving biomarker identification, therapeutic target discovery, and personalized treatment strategies.

## 12 Limitations

Despite providing a comprehensive overview of colorectal cancer pathogenesis, diagnostic strategies, and emerging artificial intelligence applications, this review has several limitations. First, rapid advancements in multi-omics technologies and computational oncology indicating that new discoveries continue to emerge, and some recent developments may not be fully captured. Second, many artificial intelligence and quantum computing approaches discussed remain in early experimental or proof-of-concept stages and require further validation in large-scale prospective clinical studies. Third, integration of multi-omics datasets presents challenges related to data heterogeneity, standardization, and computational complexity, which may affect reproducibility and clinical translation. Additionally, while AI models demonstrate high predictive accuracy, their clinical implementation may be limited by data availability, interpretability, and regulatory considerations. Addressing these limitations will be essential for successful translation of computational oncology approaches into routine clinical practice.

## 13 Future perspectives

Future research in colorectal cancer should focus on integrating multi-omics technologies, artificial intelligence, and precision oncology approaches to improve early detection, prognostic assessment, and therapeutic decision-making. Single-cell sequencing and spatial transcriptomics will provide deeper insights into tumor heterogeneity, immune interactions, and therapeutic resistance mechanisms. Artificial intelligence-driven predictive models integrating genomic, transcriptomic, imaging, and clinical data have the potential to improve personalized treatment strategies and identify novel therapeutic targets.

Furthermore, prospective clinical validation of AI-based diagnostic and prognostic tools will be critical for clinical implementation. Advances in quantum computing and hybrid computational models may enhance biomarker discovery, drug design, and predictive modeling by enabling analysis of complex biological datasets with greater efficiency. Future studies should also focus on improving model interpretability, data standardization, and multi-institutional collaboration to facilitate clinical translation and improve patient outcomes.

## 14 Conclusion

This review consolidates key insights into colorectal carcinogenesis, emphasizing the intricate interplay of genetic, epigenetic, environmental, and lifestyle factors. In parallel, the integration of artificial intelligence (AI) and machine learning (ML) with multi-omics platforms has redefined how molecular complexity and tumor heterogeneity are interpreted in colorectal cancer. Our review highlights three major molecular pathways - chromosomal instability (CIN), microsatellite instability (MSI), and CpG island methylator phenotype (CIMP) as pivotal drivers of tumor initiation and progression. Recent AI-based models incorporating genomic, transcriptomic, and histopathological data are enhancing the classification accuracy of these molecular subtypes and improving clinical decision support systems in precision oncology. Lifestyle-associated risks, such as obesity, dietary habits, and physical inactivity, significantly influence disease incidence, while emerging evidence underscores the prognostic relevance of molecular subtyping in guiding treatment selection. Advances in non-invasive diagnostics, including fecal immunochemical testing (FIT) and novel biomarkers, offer promising avenues for early detection. Furthermore, the review underscores the therapeutic potential of precision oncology, integrating molecular profiling with tailored treatment regimens, as well as the importance of primary, secondary, and tertiary preventive strategies including chemoprevention, vitamin D supplementation, and lifestyle modification in reducing disease burden and improving survival. The emergence of AI-assisted radiogenomics, spatial transcriptomics, and quantum machine learning offers transformative potential for non-invasive diagnostics, drug repurposing, and predictive modeling of treatment response in CRC. These innovations are expected to accelerate translational research and strengthen precision-based therapeutic design. Collectively, these findings advocate for a holistic, precision-based approach to colorectal cancer management, combining prevention, early diagnosis, and individualized therapy to curb its global impact.
